# ASF1B is a Promising Prognostic Biomarker and Correlates With Immunotherapy Efficacy in Hepatocellular Carcinoma

**DOI:** 10.3389/fgene.2022.842351

**Published:** 2022-03-10

**Authors:** Shirong Zhang, Longwen Xu, Jinteng Feng, Deli Tan, Yue Zhu, Jia Hou, Wenyuan Li, Kejia Lv, Wenjuan Wang, Lili Jiang, Min Jiao, Hui Guo

**Affiliations:** ^1^ Department of Medical Oncology, The First Affiliated Hospital of Xi’an Jiaotong University, Xi’an, China; ^2^ Key Laboratory of Environment and Genes Related to Diseases, Ministry of Education of China, Xi’an Jiaotong University, Xi’an, China; ^3^ Bioinspired Engineering and Biomechanics Center (BEBC), Xi’an Jiaotong University, Xi’an, China

**Keywords:** Hepatocellular carcinoma, ASF1B, prognosis, immune microenvironment, cancer immunotherapy

## Abstract

**Background:** Anti-silencing function 1B (ASF1B), a histone H3-H4 chaperone, is crucial for S-phase progression and cell proliferation. Recent studies have shown that ASF1B may be used as a new proliferation marker for cancer prognosis. However, the prognostic value and effect of ASF1B on tumor cells and the immune microenvironment in hepatocellular carcinoma (HCC) remain unclear.

**Methods:** We analyzed the expression of ASF1B and its prognostic value using The Cancer Genome Atlas (TCGA) database (as a training set) and other databases, and we validated the findings by immunohistochemistry in our clinical database, containing 141 HCC patients (as a validation set). Gene set enrichment analysis (GSEA) and gene set variation analysis (GSVA) were performed to probe the tumor-associated biological processes of ASF1B in HCC. The interrelationships between ASF1B expression and tumor immunological characteristics were analyzed by multiple databases. The Imvigor210 cohort was retrieved to assess the ability of ASF1B to predict immunotherapy efficacy.

**Results:** ASF1B was highly expressed in tumor tissue compared to paracancerous tissue. High ASF1B expression was associated with worse overall survival (OS) and progression-free survival (PFS) in the training set (*p* = 0.005, *p* < 0.001) and validation set (*p* < 0.001, *p* < 0.001). Multivariate analysis revealed that ASF1B was an independent prognostic factor associated with OS and PFS. GSEA and GSVA suggested that ASF1B was involved in tumor-associated biological processes, including the cell cycle, DNA replication, base excision repair, mismatch repair, RNA degradation, ubiquitin-mediated proteolysis, and nucleotide excision repair. Further analysis revealed that the levels of ASF1B were positively correlated with the immune cells infiltration of B cells, CD8^+^ T cells, CD4^+^ T cells, neutrophils, and dendritic cells. However, ASF1B was positively correlated with Treg cell infiltration and inhibitory immune checkpoints in exhausted T cells. Patients who received anti-PD-L1 immunotherapy with high ASF1B expression had a higher objective response.

**Conclusion:** The ASF1B level is an independent prognostic factor and may serve as a potential immunotherapeutic target.

## Introduction

Hepatocellular carcinoma (HCC), the major type of primary liver cancer, is estimated to be the fourth most common cause of cancer-related death, which exacts a heavy disease burden worldwide (Llovet et al., 2021). China is one of the highest burden areas for HCC due to the high prevalence of chronic hepatitis B virus infection, which is the most prominent risk factor for HCC development (Yu et al., 2014; Akinyemiju et al., 2017; Liu et al., 2018). Although advancements in the clinical management of HCC have improved patient survival, the prognosis of HCC remains poor due to the high rate of frequent recurrence and intrahepatic metastasis. Recently, many studies have demonstrated that immune checkpoint inhibitors (ICIs), including atezolizumab, nivolumab, and pembrolizumab, are revolutionizing cancer therapy in HCC by inducing durable antitumor responses and overall survival benefits (El-Khoueiry et al., 2017; Zhu et al., 2018; Finn et al., 2020; Yau et al., 2020). However, only a minority of patients achieve this transcendent, durable benefit from ICIs. Thus, identifying a biomarker to predict prognosis and response to ICIs for HCC treatment is urgently required.

The tumor immune microenvironment (TIME) is a crucial factor for the progression of HCC and for the response to immunotherapy (Cariani and Missale, 2019). Over the past decade, emerging evidences have revealed crosstalk between the tumor cell cycle and the TIME (Petroni et al., 2020). Disrupting cell cycle progression through targeting cell cycle regulators modulates the expression of checkpoint molecules and influences immune cell populations of the TIME, ultimately improving the efficacy of ICIs (Goel et al., 2017; Deng et al., 2018; Greten and Korangy, 2018; Zhou et al., 2018; Teh and Aplin, 2019). For example, programmed cell death-ligand 1 (PD-L1) expression fluctuates during the cell cycle, and inhibition of cyclin-dependent kinase 4 and 6 (CDK4/6) increases the level of PD-L1 through multiple pathways (Goel et al., 2017; Zhang H et al., 2018; Jin et al., 2019). In addition, cell cycle-related kinase inhibition reduces the accumulation of myeloid-derived suppressor cells (MDSCs) and improves antitumor immunity (Zhou et al., 2018). Inhibition of CDK4/6 markedly suppresses the proliferation of CD4^+^ FOXP3^+^ regulatory T cells (Tregs) and enhances CD8^+^ T cell activity (Goel et al., 2017; Deng et al., 2018). CDK7 inhibition induces a significant increase in the percentage of total T cells, natural killer cells, dendritic cells, monocytes, and neutrophils (Zhang H. et al., 2020). Therefore, targeting cell cycle regulatory proteins may be a breakthrough point to sensitize tumors to immunotherapy.

In all eukaryotes, DNA and histones are precisely organized into chromatin, and assembly and disassembly processes are vital processes during the cell cycle. Histone chaperones are involved in all aspects of histone dynamics, particularly promoting specific chromatin assembly pathways throughout cellular life (De Koning et al., 2007; Eitoku et al., 2008). Anti-silencing function 1 (ASF1), which is the most conserved H3-H4 chaperone, is crucial for S-phase progression, and it has been implicated in gene replication, transcription, and DNA repair. ASF1 exists in two paralogs, termed ASF1A and ASF1B (Natsume et al., 2007). Although conservation core-binding domain for histones H3-H4 in these paralogs suggests common properties, in fact they are not functionally equivalent. ASF1A contributes mainly to DNA repair and cell senescence, while ASF1B is preferentially involved in cell cycle progression and cell proliferation (Corpet et al., 2011; Jiangqiao et al., 2019). In addition to affecting the tumor cell intrinsic features, it is reported that ASF1B can also have an impact on the tumor microenvironment by promoting the infiltration of immune cells (Zhan et al., 2021). It has also been reported that high ASF1B expression is closely related to poor outcomes of patients with renal cell cancer, cervical cancer, and breast cancer (Corpet et al., 2011; Han et al., 2018; Jiangqiao et al., 2019; Liu et al., 2020). Therefore, ASF1B is gaining attention as a new diagnostic and prognostic biomarker as well as a therapeutic target for these cancers. However, there are no comprehensive reports about the expression and prognostic value of ASF1B and its correlation with tumor immunity in HCC.

In the present study, we systematically and comprehensively analyzed the expression of ASF1B from online public databases and our medical center’s databases and then assessed its correlation with clinicopathological factors and patients prognosis using data from The Cancer Genome Atlas database (TCGA) and the database from our center. We further explored the potential associations of ASF1B with tumor-infiltrating immune cells and the efficacy of immunotherapy using multiple online public databases.

## Materials and Methods

### Patients and Sample Collection

In total, 141 patients were enrolled from January 2013 to December 2015 as a validation set to validate the findings from public databases. Sixty-one of these patients had paired tumors and adjacent tissue samples. All patients in this cohort underwent R0 resection, and the pathological diagnosis was confirmed as HCC identified by the pathology department at the First Affiliated Hospital of Xi’an Jiaotong University (Xi’an, China). The patients’ primary characteristics are listed in Table 1. All patients were followed up until December 2020. The use of human tissues in this study was approved by the Research Ethics Committees.

**TABLE 1:** Demographics and clinicopathological characteristics of the HCC patients in TCGA database and our Center.

Features	Training set (*n* = 371)	Validation set (*n* = 141)
Age [years, median (range)]	59.4 (16–90)	51.0 (27–80)
Sex (Female/Male) (%)	121/250 (32.6/67.4)	22/119 (15.6/84.4)
HBV (Positive/Negative) (%)	—	123/18 (87.2/12.8)
AFP (ng/ml, <400/≥400/unknown) (%)	213/65/93 (57.4/17.5/25.1)	90/51 (63.8/36.2)
Tumor number (Single/Multinodular) (%)	—	98/43 (69.5/30.5)
Tumor differentiation (Edmondson-Steiner grade) (I/II/III/IV/unknown) (%)	55/177/122/12/5 (14.8/47.7/32.9/3.2/1.3)	7/91/43/0/0 (5.0/64.5/30.5/0/0)
Tumor diameter (cm, mean ± SD)	—	7.27 ± 7.26
Vascular invasion (yes/no/unknown) (%)	206/109/56 (55.5/29.4/15.1)	93/48 (66.0/34.0)
T classification (T1/T2/T3/T4/unknown) (%)	181/94/80/13/3 (48.8/25.3/21.6/3.5/0.8)	63/56/19/3 (44.0/39.7/13.5/2.1)
N classification (N0/N1/unknown) (%)	252/4/115 (67.9/1.1/31.0)	135/6 (95.7/4.3)
M classification (M0/M1/unknown) (%)	266/4/101 (71.7/1.1/27.2)	—
TNM stage (I/II/III/IV/unknown) (%)	171/86/85/5/24 (46.1/23.2/22.9/1.3/6.5)	60/55/21/5 (42.6/39.0/14.9/3.5)
ASF1B expression (High/Low) (%)	185/186 (49.9/50.1)	45/96 (31.9/68.1)

HBV, hepatitis B virus; AFP, alpha fetoprotein; TNM, tumor node metastasis.

### Immunohistochemistry and Evaluation of Immunostaining

Immunohistochemistry was performed as described previously (Zhang et al., 2019). The ASF1B antibody (human, diluted 1:200, ab235358, Abcam, Cambridge, MA) was applied. The tumor cells in which nuclei were stained dark brown under light microscopy were considered positive. The staining intensity was evaluated with the following scoring system: 0 point represented no staining; 1 point represented weak staining intensity; 2 point represented moderate staining intensity; and 3 point represented strong staining intensity. Additionally, the percentage of stained tumor cells was assessed as follows: 0 point indicated 0%; 1 point indicated less than 25%; 2 point indicated 25–50%; and 3 point indicated more than 50%. The final score was equal to the multiplication of the above two scores. A score of 0–3 point represented a low expression level of ASF1B, while a score greater than 3 point indicated high expression (Liu et al., 2020).

### Cell Culture, RNA Interference and Transfection

Hep3B and Huh7 were a gift from JuSeog Lee (MD Anderson Cancer Center, Houston, TX). All cells were maintained in high glucose Dulbecco’s modified Eagle’s medium (Hyclone, Logan, UT) with 10% FBS (Hyclone, Logan, UT) and 100 U/ml penicillin and streptomycin. Cell lines were incubated with 95% humidified air and 5% CO_2_ at 37°C. The human ASF1B-target small interfering (si)RNAs (siRNA1: 5′-3′ CAG​GCG​GGA​AUG​UUA​GUU​ATT, 3′-5′ UAA​CUA​ACA​UUC​CCG​CCU​GTT; siRNA2: 5′-3′ CAU​GUU​GCC​UUU​CCU​GUC​ATT, 3′-5′ UGA​CAG​GAA​AGG​CAA​CAU​GTT) were applied to construct ASF1B knockdown HCC lines. In this process Lipofectamine 2000 (Invitrogen, MA) was used according to it’s protocol.

### MTT Analysis and Colony Formation Assay

HCC cells (5,000 per well) were seeded into 96-well plates. Relative cell numbers were quantified per day using the 3-(4,5-dimethylthia-zol-2-yl)-2,5-diphenyltetrazolium bromide (MTT) assay. Absorbance was measured at 492 nm. For colony formation assays, HCC cells were seeded in the 6-well plates at a density of 500/well. After 14 days, Each group were fixed in 4% paraformaldehyde for 20 min and stained with 0.1% crystal violet solution for imaging and counting.

### Western Blotting Analysis

At first, protein was extracted from LO2 normal liver cells and 5 HCC cell lines (Hep3B, SMMC7721, MHCC97L, MHCC97H, and Huh7). Then Western blotting was operated in accordance with standard protocols. The following primary antibodies were applied: anti-β-actin antibody (human, diluted 1:10,000, 60004-1-Ig, Proteintech Group, United States) and anti-ASF1B antibody (diluted 1:1,000). The ASF1B detection was repeated at least three times. The band intensities were measured using ImageJ (Bethesda, MD, United States).

### Collection of Sample Information From TCGA

Clinical information and transcriptomic data of 375 HCC patients and 49 adjacent cancer samples were downloaded from TCGA data portal (https://portal.gdc.cancer.gov/). Cases with insufficient or missing data were filtered out, and 371 patients data were adopted as a training set for survival analysis. We compared the overall survival (OS) and progression-free survival (PFS) of HCC patients divided by the ASF1B median expression value. The clinical characteristics of the included patients were summarized in Table 1.

### HCCDB Data Analysis

The Integrative Molecular Database of Hepatocellular Carcinoma (HCCDB) database (http://lifeome.net/database/hccdb) was used to explore ASF1B mRNA expression in HCC and adjacent noncarcinoma tissues. The HCCDB database contains 15 public datasets that cover 3,917 samples (Lian et al., 2018).

### Heterogeneity

Profile of somatic mutation data of the TCGA-LIHC cohort were obtained from the GDC portal on February 13, 2022. With the advantage of R “maftools” package, we visualized the MAF files of simple nucleotide variation which processed by the workflow type of varScan2 variant aggregation and masking. The tumor mutation burden (TMB) and the mutant-allele tumor heterogeneity (MATH) score of tumor samples in the TCGA-LIHC dataset were also computed via the “maftools” package.

### Gene Set Enrichment Analysis and Gene Set Variation Analysis

Gene set enrichment analysis (GSEA) and gene set variation analysis (GSVA) were used to investigate the mechanisms of ASF1B in HCC (Hu et al., 2021; Lin et al., 2021). In the present study, the enrichment scores (ES) of “c2.cp.kegg.v6.2.symbols.gmt’’ gene sets from the Molecular Signatures Database (MSigDB) in each group were counted by GSEA software (4.1.0) and reflected the degree to which a given gene set was represented in a ranked list of genes. A nominal *p* value of <0.05 and a FDR q-value of <0.25 were used as the cutoff criteria. In addition, GSVA was performed to further analyze the difference in pathways between the subtypes of ASF1B using the GSVA package (Subramanian et al., 2005; Hu et al., 2021).

### Immune Cell Infiltration Analysis

The tumor immune estimation resource (TIMER) (https://cistrome.shinyapps.io/timer/) was used to analyze immune infiltration in HCC (Li et al., 2017). We used this tool to explore the correlation between ASF1B and the abundances of six immune infiltrates (B cells, CD4^+^ T cells, CD8^+^ T cells, neutrophils, macrophages, and dendritic cells) and gene markers of infiltrating immune cells in HCC. Furthermore, other algorithms (including XCELL, QUANTISEQ, EPIC, CIBERSORT-ABS and CIBERSORT) were utilized to quantify the infiltrating immune cells and to verify the TIMER results.

### Single-Sample Gene Set Enrichment Analysis Analysis

The single-sample gene set enrichment analysis (ssGSEA) was utilized to calculated the infiltrating score of 16 immune cells and the activity of 13 immune-related pathways in the “gsva” R package (Lei et al., 2020), which aided in determining the activity of immune cells and immune pathways of each sample.

### Statistical Analyses

In this study, unpaired t test and one-way analysis of variance were used to calculate the comparison of continuous variables. The chi-square test was used to process the categorical variables. Kaplan-Meier survival curves was used to display the OS and PFS. Univariate and multivariate regression analyses were used to identify independent prognostic factors. The correlation between genes was detected with Pearson analysis. All the tests were two-sided, and *p* < 0.05 was considered statistically significant. SPSS 24.0 (SPSS Inc., Chicago, IL, United States) was used to perform the statistical analyses.

## Results

### Anti-Silencing Function 1B has Higher Expression in Hepatocellular Carcinoma Tissue Than in Adjacent Noncancerous Tissues

**FIGURE 1:**
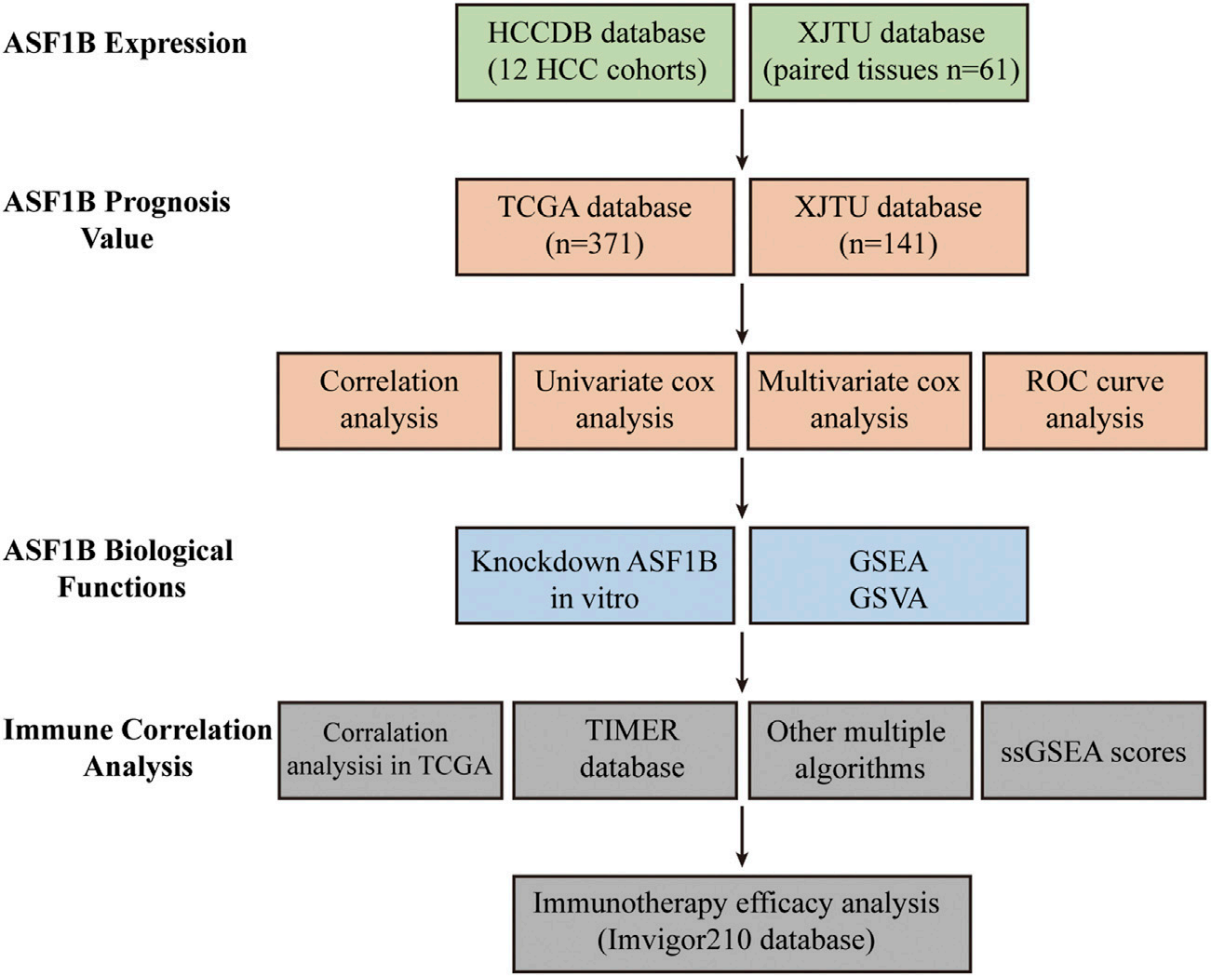
Flow chart of the study design.

**FIGURE 2:**
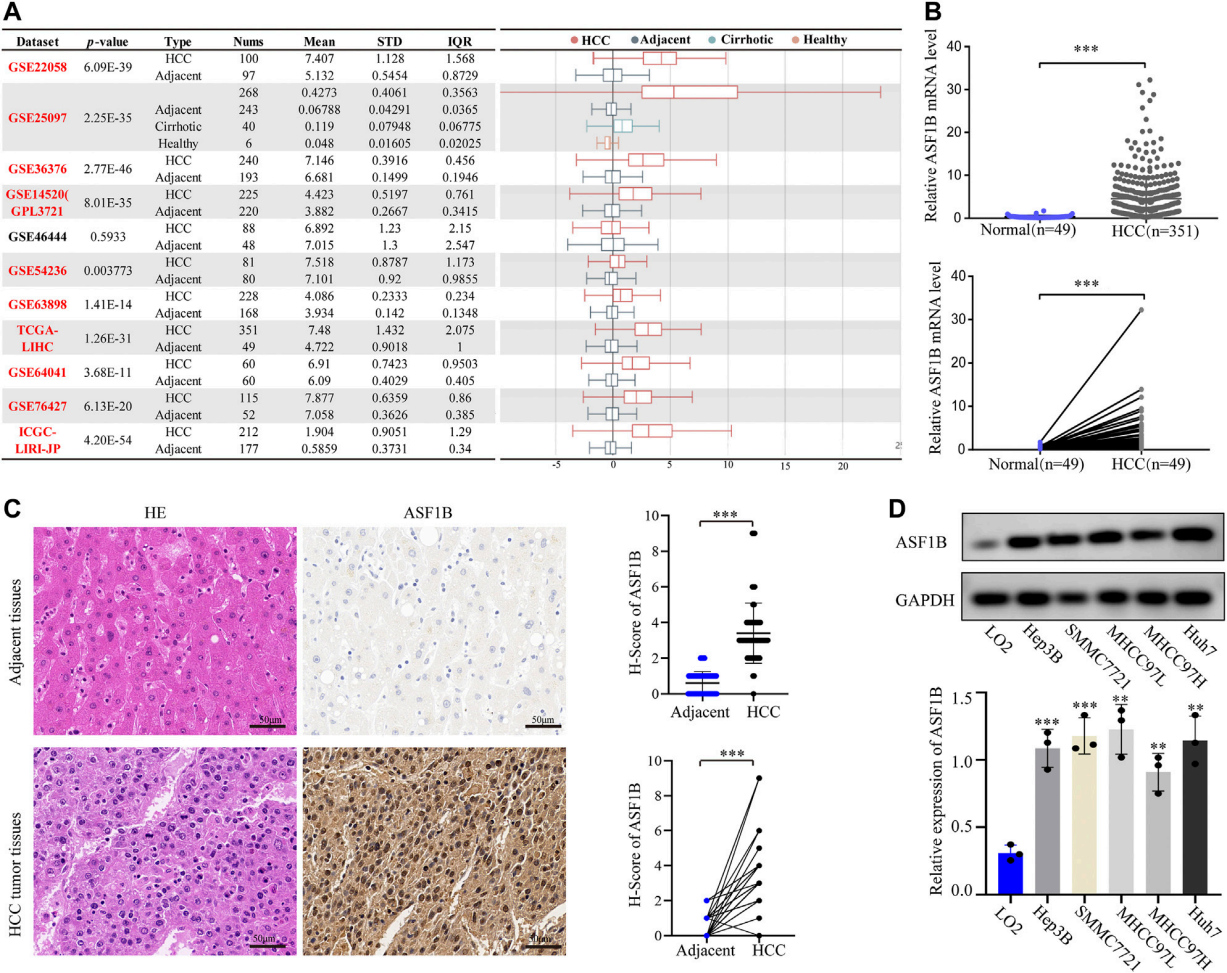
ASF1B mRNA and protein expression in HCC. **(A)** Chart and plot of ASF1B expression in HCC and matched adjacent noncancerous tissues based on HCCDB database; **(B)** Significant differences of ASF1B mRNA level in HCC and the adjacent noncancerous tissues in TCGA database; **(C)** HE images of HCC tumor tissues and adjacent noncancerous tissues (left); IHC images and H-score of the ASF1B proteins expression in HCC tumor tissues and adjacent noncancerous tissues (right). Magnification = ×400. The positive staining appears brown; **(D)** The protein expression of ASF1B in normal liver cell and HCC cells by western blot. **p < 0.01; ***p < 0.001.

A flowchart of this study is illustrated in Figure 1. An analysis of 12 HCC cohorts using the HCCDB database showed that ASF1B mRNA expression was remarkably increased in HCC relative to adjacent noncancerous tissues or cirrhotic tissues in 11 HCC cohorts (Figure 2A). Consistently, comparison of ASF1B mRNA expression in cancer and matched paracancerous tissues from TCGA database demonstrated the upregulation of ASF1B in cancer (Figure 2B).

To validate the above findings, HCC cancer tissues and corresponding adjacent tissues from 61 patients were tested by IHC. The protein expression of ASF1B in cancer tissues was higher than that in noncancerous tissues (Figure 2C). Western blotting was then performed to detect ASF1B protein expression in one normal liver cell line (LO2) and five HCC cancer cell lines (Hep3B, SMMC7721, MHCC97L, MHCC97H, and Huh7). Compared to normal hepatocytes, ASF1B was remarkably increased in the HCC cell lines (Figure 2D). Together, these data illustrated that ASF1B has higher expression in HCC tissue than in adjacent noncancerous tissues.

### High Anti-Silencing Function 1B Expression is Associated With Poor Prognosis in Hepatocellular Carcinoma

**FIGURE 3:**
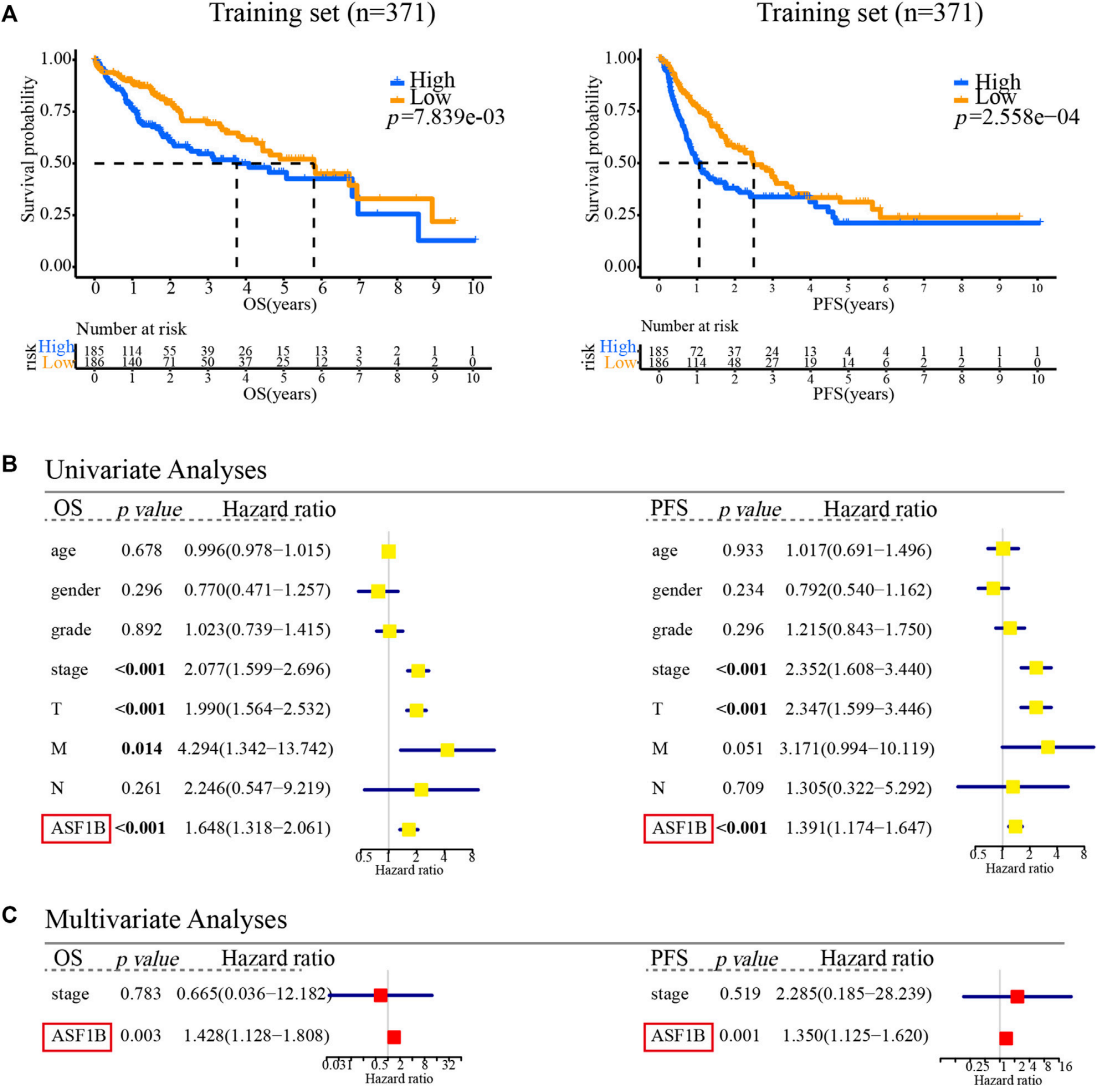
Multifaceted prognostic value of ASF1B in the training set. **(A)** Kaplan-Meier estimates of OS and PFS according to the level of ASF1B mRNA among TCGA LIHC cases. Training set, OS (*p* < 0.001), PFS (*p* < 0.001); **(B)** Univariate Cox regression analyses of OS and PFS related factors among TCGA LIHC cases; **(C)** Multivariate Cox regression analyses of OS and PFS related factors among TCGA LIHC cases.

**FIGURE 4:**
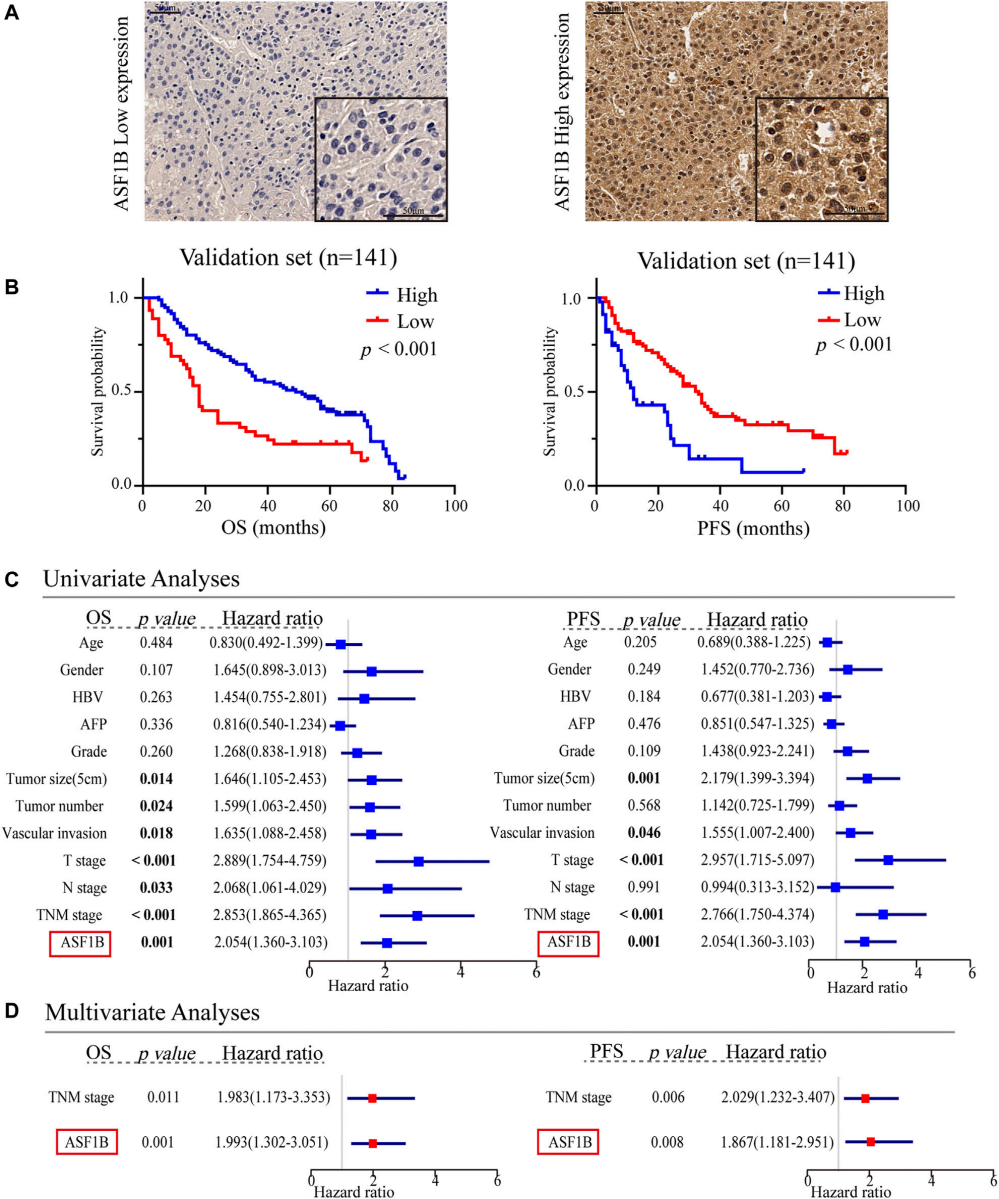
Multifaceted prognostic value of ASF1B in the validation set. **(A)** Representative microphotographs of ASF1B staining in high and low expression groups; **(B)** Kaplan-Meier estimates of OS and PFS according to the level of ASF1B protein expression through IHC in the patients with HCC in our center. Validation set, OS (*p* < 0.001), PFS (*p* < 0.001); **(C)** Univariate Cox regression analyses of OS and PFS related factors among our center HCC cases; **(D)** Multivariate Cox regression analyses of OS and PFS related factors among our center HCC cases.

To explore the prognostic value of ASF1B in HCC, we enrolled two cohorts. The training set was obtained from TCGA database and contained 371 individuals, and the validation set contained 141 HCC patients from our center who had pathology and complete follow-up information. The relationships between ASF1B expression and the clinical features of HCC patients are listed in Table 2 and Supplementary Figure S1. In the training set, a high ASF1B mRNA level was correlated with older patients (*p* < 0.001), males (*p* = 0.018), poorer differentiation (*p* < 0.001), higher levels of AFP (*p* < 0.001), more advanced T stage (*p* = 0.001), and TNM stage (*p* = 0.001). In the validation set, a high ASF1B protein level was correlated with poorer differentiation (*p* = 0.038), more advanced T stage (*p* = 0.013), and TNM stage (*p* = 0.002). The Kaplan-Meier survival curves revealed that patients with high ASF1B expression had poorer OS and PFS than patients with low ASF1B expression in the training set (*p* = 0.005, *p* < 0.001; Figure 3A) and the validation set (*p* < 0.001, *p* < 0.001; Figures 4A,B).

**TABLE 2:** The relationships between ASF1B expression and patients’ clinical features in TCGA database and our Center.

Features	Training set (*n* = 371)			Validation set (*n* = 141)		
	Low	High	*p* value	Low	High	*p* value
Age			**<0.001**			0.159
≥65 years	93	56		20	5	
<65 years	93	128		76	40	
Sex			**0.018**			0.992
Male	136	114		81	38	
Female	50	71		15	7	
HBV						0.497
Positive	—	—	—	85	38	
Negative	—	—	—	11	7	
AFP (ng/ml)			**<0.001**			0.917
≥400	20	45		35	16	
<400	122	91		61	29	
Tumor number						0.616
Single	—	—	—	68	30	
Multinodular	—	—	—	28	15	
Tumor differentiation			**<0.001**			**0.038**
I + II	139	93		72	26	
III + IV	44	90		24	19	
Vascular invasion			0.260			0.903
Yes	52	57		33	15	
No	112	94		63	30	
T classification			**0.001**			**0.013**
T1 + T2	150	125		86	33	
T3 + T4	33	60		10	22	
N classification			0.624			0.384
N0	121	131		93	42	
N1	1	3		3	3	
TNM stage			**0.001**			**0.002**
I + II	142	115		85	30	
III + IV	31	59		11	15	

HBV, hepatitis B virus; AFP, alpha fetoprotein; TNM, tumor node metastasis. The bold values means *p* < 0.05.

### Anti-Silencing Function 1B Expression is an Independent Prognostic Factor for Hepatocellular Carcinoma Patients

Besides, univariate and multivariate Cox regression analyses were performed to investigate whether ASF1B has a clinically independent prognostic value. Univariate Cox regression analysis revealed the association between OS and T stage (*p* < 0.001), M stage (*p* = 0.014), TNM stage (*p* < 0.001), and ASF1B expression (*p* < 0.001) as well as the association between PFS and T stage (*p* < 0.001), TNM stage (*p* < 0.001), and ASF1B expression (*p* < 0.001) in the training set (Figure 3B). In the validation set, more clinical factors were included for analysis. The factors significantly associated with OS were tumor size (5 cm) (*p* = 0.014), tumor number (*p* = 0.024), vascular invasion (*p* = 0.018), T stage (*p* < 0.001), N stage (*p* = 0.033), TNM stage (*p* < 0.001), and ASF1B expression (*p* < 0.001). The factors significantly associated with PFS were tumor size (5 cm) (*p* = 0.001), vascular invasion (*p* = 0.046), T stage (*p* < 0.001), TNM stage (*p* < 0.001), and ASF1B expression (*p* < 0.001) in the validation set (Figure 4C).

The variables demonstrating significance for the prognosis of HCC patients were included in the multivariate analysis. The analysis showed that ASF1B expression was an independent prognostic factor that was associated with OS (*p* = 0.003, HR = 1.428) and PFS (*p* = 0.001, HR = 1.350) in the training set. In addition, the validation set verified that ASF1B expression was indeed an independent risk factor for patient prognosis (OS: *p* = 0.001, HR = 1.993; PFS: *p* = 0.008, HR = 1.869) (Figures 3C, 4D). Further, the receiver operating characteristic (ROC) curve showed that ASF1b expression had good predictive efficacy in the training set and the validation set (OS: 0.684, 0.697; PFS: 0.673, 0.703) (Supplementary Figure S2). Thus, these findings suggested that ASF1B may serve as a valuable predictive factor for HCC patients.

### Analysis of the Biological Functions of Anti-Silencing Function 1B in Hepatocellular Carcinoma

**FIGURE 5:**
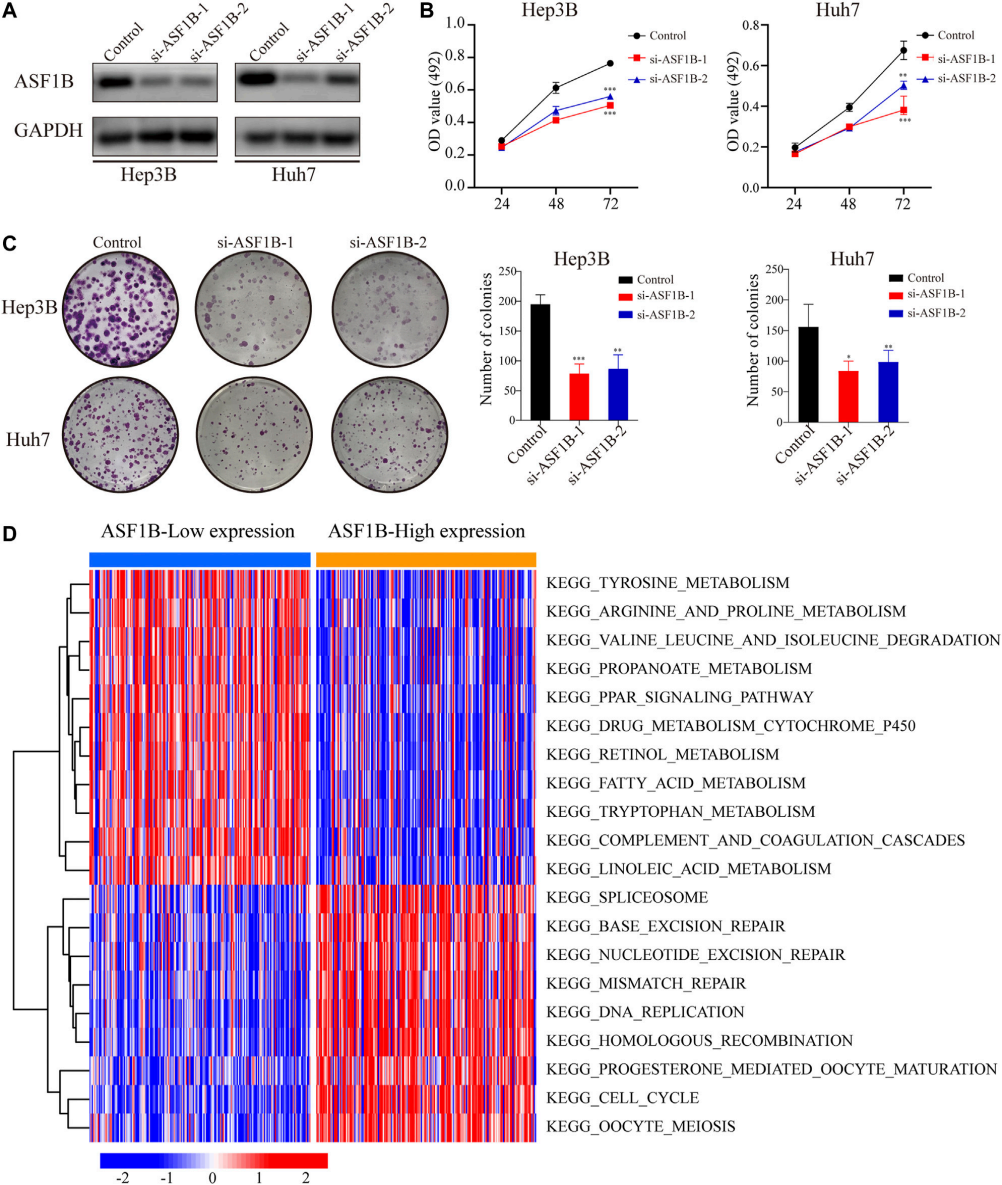
Downregulation of ASF1B inhibits the proliferation of HCC cells. **(A)** The protein levels of ASF1B were detected using Western blotting after transfection of two independent sets of ASF1B-targeted siRNAs in Hep3B and Huh7; The proliferation of HCC cells was detected by **(B)** MTT assay and **(C)** the colony formation assay; **(D)** Functional enrichment analysis of ASF1B-correlated genes in HCC through GSVA. The red node represents up-regulation and the blue node represents down-regulation. *p < 0.05; **p < 0.01; ***p < 0.001.

Previous studies have reported that ASF1B functions as an oncogene to promote tumor growth by participating in the cell cycle (Corpet et al., 2011; Han et al., 2018; Jiangqiao et al., 2019; Liu et al., 2020). Given the clinical significance of ASF1B, we further analyzed the effects of ASF1B on the biological behaviors of the HCC cells. HCC cell lines with downregulation of ASF1B were constructed (Figure 5A). Inhibition of ASF1B significantly inhibited the proliferation and colony formation of Hep3B and Huh7 (Figures 5B,C). Then GSEA and GSVA were conducted to explore the biological functions of ASF1B in HCC. GSEA suggested that the ASF1B high-expression phenotype was mainly involved in the cell cycle in HCC. Apart from this, ASF1B was also related to DNA replication, the spliceosome, base excision repair, oocyte meiosis, homologous recombination, mismatch repair, RNA degradation, ubiquitin-mediated proteolysis, and nucleotide excision repair (Table 3). In addition, GSVA confirmed that cell cycle pathways were significantly differentially expressed between the high ASF1B group and the low ASF1B group in HCC. The heatmap shown in Figure 5D displays the differential expression of specific signaling pathways. Overall, these results demonstrated that ASF1B is mainly correlated with dysregulation of the cell cycle process, which may promote proliferation and be involved in the poor prognosis of HCC patients.

**TABLE 3:** Gene sets enriched in the ASF1B high-expression phenotype in HCC.

Gene set name	NES	NOM *p*-value	FDR q-value
KEGG_CELL_CYCLE	2.2	<0.001	0.004
KEGG_SPLICEOSOME	2.16	<0.001	0.005
KEGG_BASE_EXCISION_REPAIR	2.1	<0.001	0.007
KEGG_DNA_REPLICATION	2.04	<0.001	0.013
KEGG_OOCYTE_MEIOSIS	2.01	<0.001	0.017
KEGG_HOMOLOGOUS_RECOMBINATION	1.97	<0.001	0.02
KEGG_MISMATCH_REPAIR	1.96	<0.001	0.024
KEGG_RNA_DEGRADATION	1.91	<0.001	0.035
KEGG_UBIQUITIN_MEDIATED_PROTEOLYSIS	1.89	<0.001	0.041
KEGG_NUCLEOTIDE_EXCISION_REPAIR	1.89	<0.001	0.038
KEGG_PROGESTERONE_MEDIATED_OOCYTE_MATURATION	1.88	<0.001	0.047

NES, Normalized Enrichment Score; FDR, false discovery rate.

### Gene Mutation in Anti-Silencing Function 1B-High and Anti-Silencing Function 1B-Low Expression Group

**FIGURE 6:**
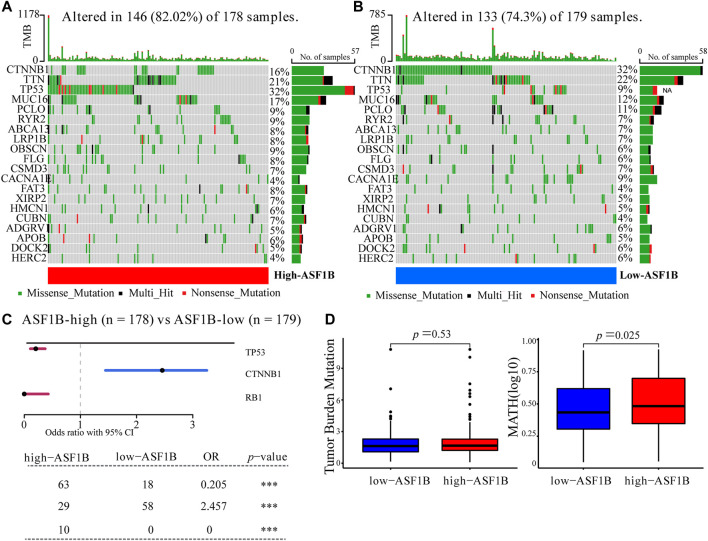
Somatic mutation between ASF1B-high and ASF1B-low expression group. Oncoplots displayed the top-20 mutated genes in the **(A)** high-ASF1B and **(B)** low-ASF1B group of the TCGA-LIHC dataset. **(C)** Forest plot revealed three mutated genes with statistic significant between the ASF1B-high and ASF1B-low group. **(D)** The comparison of TMB (left) and MATH (right) scores in the ASF1B-high and ASF1B-low group. ***p < 0.001.

To explore the difference of genomics between the ASF1B-highand ASF1B-low subsets, we examined somatic mutation from the TCGA-LIHC datasets. Figure 6A showed that the top-5 highest mutation prevalence genes in the ASF1B-high group were TP53 (32%), TTN (21%), MUC16 (17%), CTNNB1 (16%), PCLO (9%), whereas those in the ASF1B-low group (Figure 6B) were CTNNB1 (32%), TTN (22%), MUC16 (12%), PCLO (11%), and TP53 (9%). We identified two genes (TP53, RB1) were highly mutated in the ASF1B-high group than in ASF1B-low group, while CTNNB1 exhibited higher mutation frequency in ASF1B-low group (Figure 6C). Although the TMB was not significantly different between ASF1B-low and ASF1B-high groups, patients from high-ASF1B group possessed obviously elevated MATH scores, suggesting a higher level of tumor heterogeneity in this group (Figure 6D, *p* = 0.025).

### Correlation Between Anti-Silencing Function 1B Expression and Immune Cell Infiltration in Hepatocellular Carcinoma

**FIGURE 7:**
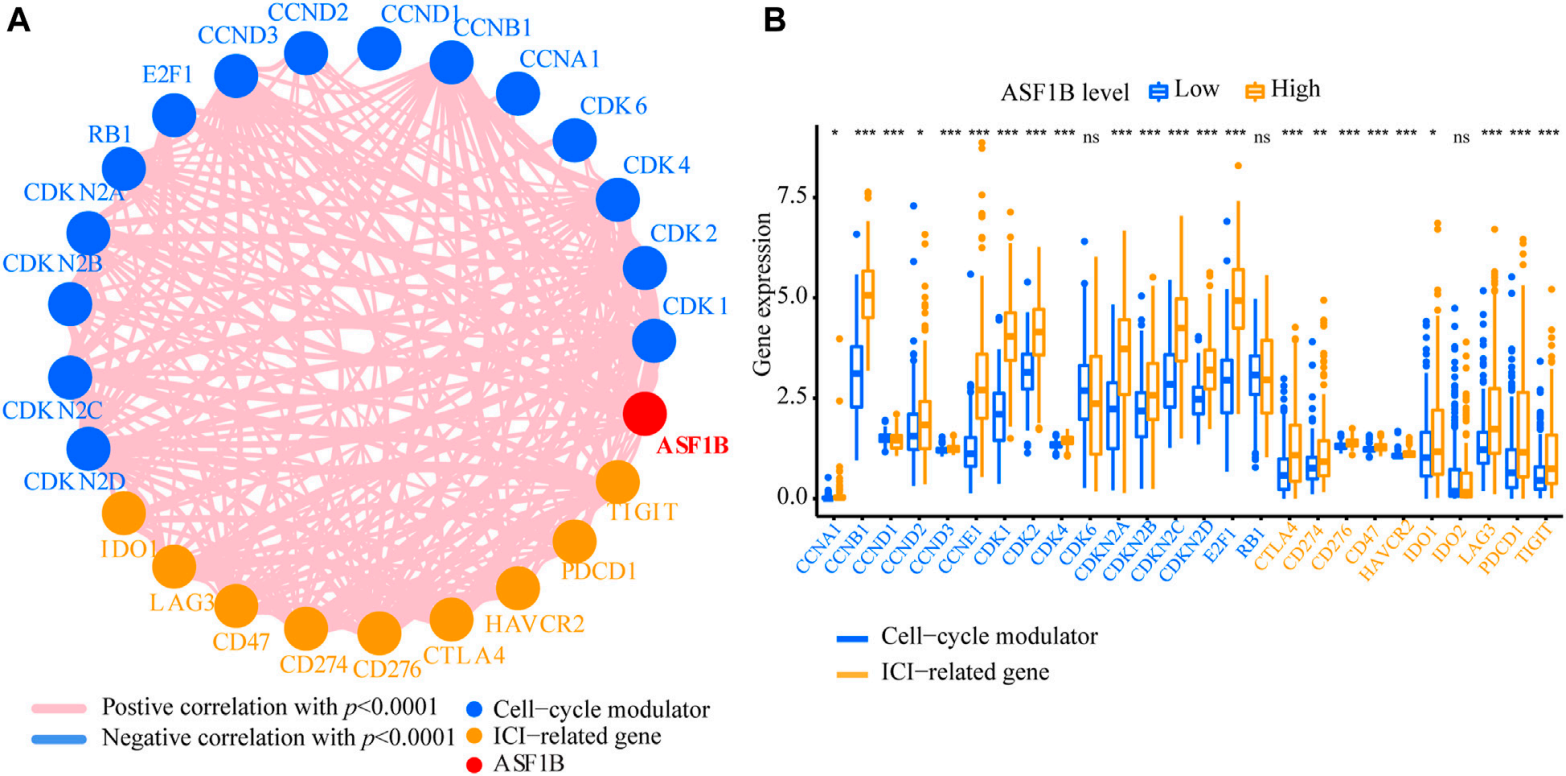
The relationship of cell-cycle modulators and immune checkpoints between ASF1B-high and ASF1B-low groups. **(A)** Correlation of ASF1B, cell-cycle modulators, and immune checkpoints. **(B)** The comparison of cell-cycle modulators and immune checkpoints between ASF1B-high and ASF1B-low groups. *p < 0.05; **p < 0.01; ***p < 0.001.

Emerging preclinical and clinical evidence has revealed that blocking the tumor cell cycle not only inhibits the proliferation of cancer cells but also mediates a wide range of immunomodulatory effects that involve both malignant and nonmalignant components of the TIME, thereby suggesting novel immunotherapeutic avenues (Petroni et al., 2020). Thus, we explored the correlation between ASF1B expression and immune cell infiltration. First, we examined the relationship of ASF1B expression with 15 core cell-cycle modulators and 10 immune checkpoints. According to the Pearson correlation analysis, the ASF1B expression level was significantly correlated with the expression of cell-cycle modulators, including CDK1, CDK2, CDK4, CDK6, CCND1 and so on. ASF1B expression was also significantly associated with immune checkpoints expression such as CD274, CTLA4 and PDCD1, etc. Meantime, cell cycle regulatory molecules were positively related to immune checkpoint molecules, further suggesting the close link between cell cycle and tumor immunity (Figure 7A). Additionally, results of Wilcoxon test showed that 13 cell-cycle modulators and nine immune checkpoints were significantly increased in the ASF1B-high group (Figure 7B). Above results validated our hypothesis that ASF1B, as an important cell cycle regulator, may affect the HCC tumor immunity.

**FIGURE 8:**
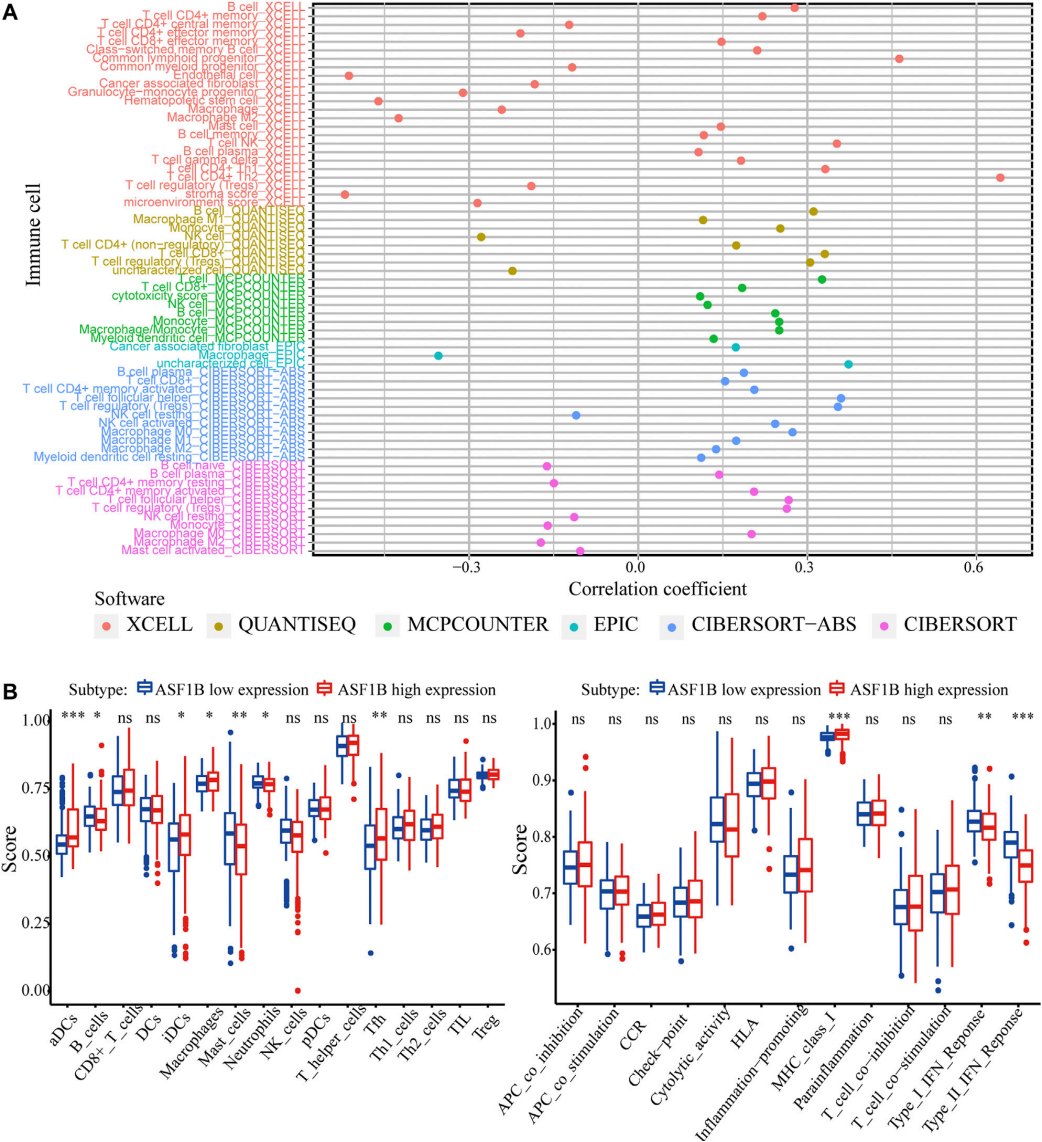
ASF1B expression is related to immune cell infiltration. **(A)** The correlation between immune cell infiltration and ASF1B expression in HCC; **(B)** Comparison of the ssGSEA scores between high and low expression groups in the TCGA cohort. *p < 0.05; **p < 0.01; ***p < 0.001.

Next, we explored the correlation between ASF1B expression and immune cell infiltration. In the TIMER database, the ASF1B expression level was significantly and positively correlated with immune cell infiltration, including CD4^+^ T cells, CD8^+^ T cells, B cells, macrophages, neutrophils, and dendritic cells (DCs) in HCC (Supplementary Figure S3). The xCell database analysis showed that ASF1B was positively related to CD4^+^ Th1 cells (*r* = 0.332, *p* < 0.001), CD4^+^ Th2 cells (*r* = 0.642, *p* < 0.001), and NK cells (*r* = 0.352, *p* < 0.001) infiltration. The QUANTISEQ results indicated that ASF1B was positively related to B cells (*r* = 0.311, *p* < 0.001), CD8^+^ T cells (*r* = 0.331, *p* < 0.001) and Treg cells (*r* = 0.305, *p* < 0.001) infiltration (Figure 8A; Supplementary Table S1). However, there were significant differences in the infiltration degrees of B cells, dendritic cells, neutrophils, macrophages, and Thf cells, but not T cells, between the high and low ASF1B expression groups according to ssGSEA (Figure 8B). Compared to the low expression group, the related functions or pathways of type I IFN response and type II IFN response were lower in the high ASF1B expression group, while the related functions or pathways of the MHC class I were higher in the high expression group. These results suggested that ASF1B is closely associated with the degree of immune cell infiltration in HCC.

**FIGURE 9:**
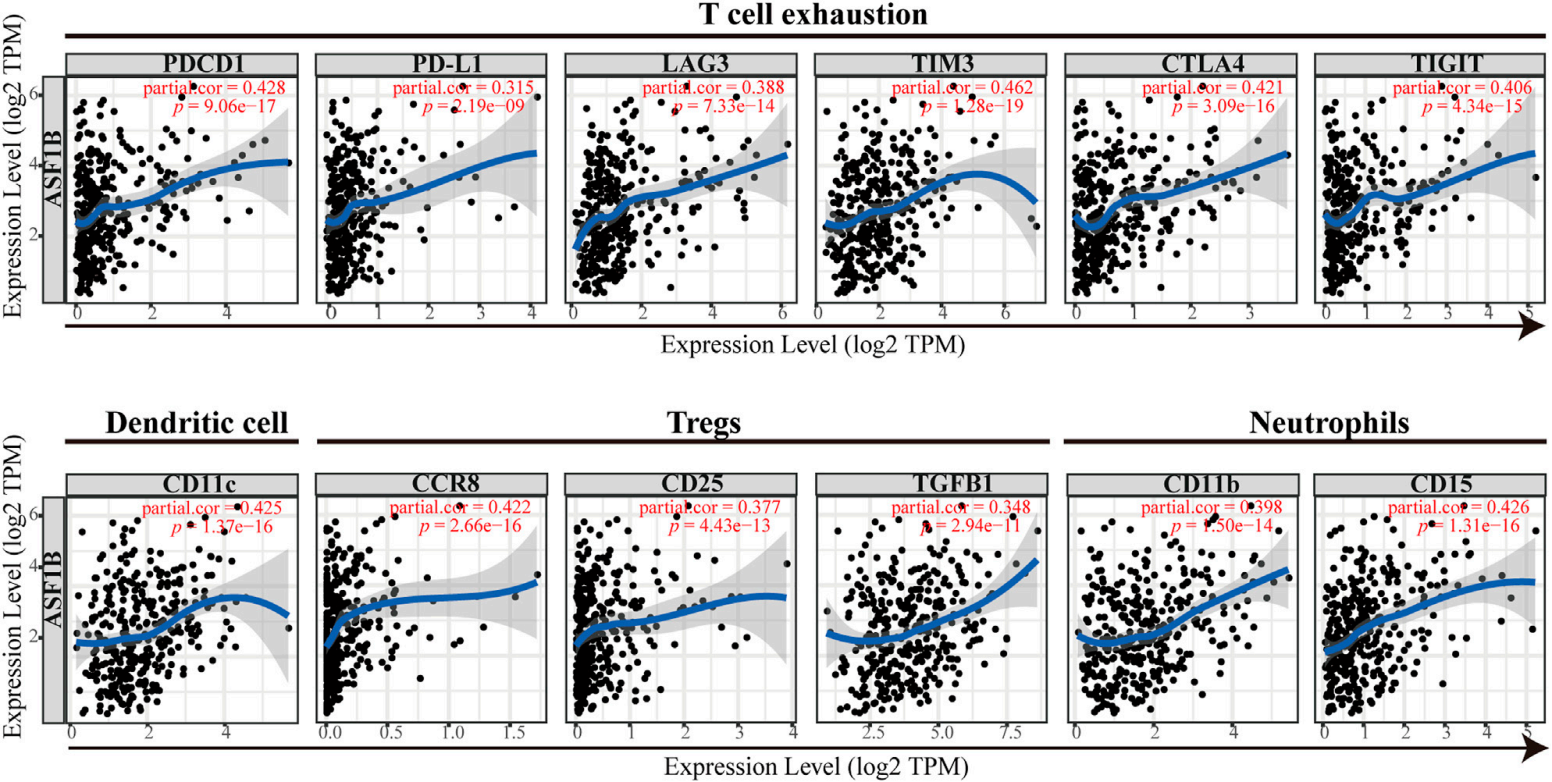
The correlation between the immune marker sets of immune cells and ASF1B expression in HCC.

To validate the above results, the correlation between ASF1B and the immune marker sets of immune cells in HCC was explored using the TIMER database. The correlation was adjusted for tumor purity, which influences the immune infiltration analysis. As shown in Figure 9 and Table 4, ASF1B expression was positively correlated with most of the immune marker sets. Interestingly, ASF1B was significantly positively correlated with inhibitory immune checkpoints in exhausted T cells, including PD-1 (*r* = 0.428, *p* = 9.06e-17), CTLA-4 (*r* = 0.421, *p* = 3.09e-16), LAG3 (*r* = 0.388, *p* = 7.33e-14), TIM-3 (*r* = 0.421, *p* = 3.09e-16), and TIGIT (*r* = 0.406, *p* = 4.34e-15). In addition, ASF1B also correlated with the CCR8, STAT5b, and TGFB1 gene markers in Tregs as well as the CD11b and CD15 gene markers in neutrophils. The above results suggested that ASF1B may indicate an immunosuppressive tumor microenvironment with increasing immune suppressive cells and dysfunctional and exhausted T cells even though it may cause increased T cell and other immune cells infiltration, which could influence the patient outcome in HCC. The hypothesis figure for the correlation between ASF1B expression and immune cell distribution is illustrated in Supplementary Figure S4.

**TABLE 4:** Correlation between ASF1B expression and immune marker sets of immune cells in HCC based data from TIMER database.

Terms	Markers	None		Tumor purity		Terms	Markers	None		Tumor purity	
		*R*	*p*-value	*R*	*p*-value			*R*	*p*-value	*R*	*p*-value
T cell exhaustion	PDCD1	0.315	5.63E-10	0.428	9.06E-17	TAM	CCL2	0.07	1.81E-01	0.195	2.74E-04
	CD274 (PD-L1)	0.228	8.81E-06	0.315	2.19E-09		CD68	0.204	7.48E-05	0.309	4.66E-09
	CTLA-4	0.299	4.17E-09	0.421	3.09E-16		IL10	0.175	6.97E-04	0.297	1.83E-08
	LAG3	0.334	3.78E-11	0.388	7.33E-14	Natural killer cell	KIR2DL1	−0.012	8.17E-01	−0.029	5.88E-01
	HAVCR2 (TIM3)	0.287	1.81E-08	0.462	1.28E-19		KIR2DL3	0.187	2.84E-04	0.238	7.85E-06
	TIGIT	0.266	1.99E-07	0.406	4.34E-15		KIR3DL1	0.02	6.97E-01	0.04	4.61E-01
	B7-H3	0.541	1.30E-29	0.551	9.32E-29		KIR3DL2	0.09	8.30E-02	0.14	9.01E-03
	B7-H4	0.242	2.29E-06	0.311	3.64E-09		CD56	0.175	7.12E-04	0.271	3.33E-07
	BTLA (CD272)	0.14	6.87E-03	0.28	1.19E-07		CD335 (NCR1)	0.032	5.37E-01	0.098	6.93E-02
	HVEM	0.327	1.03E-10	0.36	5.15E-12	Neutrophils	CD66b (CEACAM8)	0.054	2.97E-01	0.088	1.01E-01
	CD96	0.176	6.49E-04	0.325	6.00E-10		CD11b (ITGAM)	0.29	1.33E-08	0.398	1.50E-14
	CD273 (PD-L2)	0.062	2.35E-01	0.193	3.01E-04		CD15	0.388	9.57E-15	0.426	1.31E-16
	GZMB	0.096	6.51E-02	0.182	6.90E-04	Monocyte	CD14	−0.394	3.07E-15	−0.353	1.56E-11
T cell (general)	CD3D	0.252	8.86E-07	0.38	2.66E-13		CD115 (CSF1R)	0.142	6.23E-03	0.3	1.25E-08
	CD3E	0.164	1.55E-03	0.332	2.54E-10	Dendritic cell	BDCA-1 (CD1C)	0.082	1.13E-01	0.184	5.90E-04
	CD2	0.183	3.98E-04	0.339	1.05E-10		BDCA-3	−0.006	2.07E-01	0.02	7.16E-01
	CD28	0.238	2.73E-06	0.335	1.78E-10		BDCA-4 (NRP1)	0.193	1.79E-04	0.225	2.56E-05
CTL (Cytotoxic T Lymphocytes )	CD8A	0.197	1.38E-04	0.323	7.60E-10		CD123	0.054	3.02E-01	0.129	1.68E-02
	CD8B	0.201	9.72E-05	0.32	1.14E-09		CD11c (ITGAX)	0.283	2.85E-08	0.425	1.37E-16
	CD45	0.206	6.59E-05	0.346	3.82E-11	Th1	T-bet (TBX21)	0.051	3.23E-01	0.169	1.61E-03
	HLA-DPB1	0.175	7.04E-04	0.317	1.82E-09		STAT4	0.242	2.42E-06	0.314	2.42E-09
	HLA-DQB1	0.148	4.39E-03	0.272	2.84E-07		STAT1	0.335	3.76E-11	0.387	8.70E-14
	HLA-DRA	0.172	8.74E-04	0.311	3.60E-09	Th2	GATA3	0.17	1.02E-03	0.318	1.55E-09
	HLA-DPA1	0.142	6.55E-03	0.285	6.83E-08		STAT6	0.127	1.43E-02	0.123	2.26E-02
B cell	CD19	0.269	1.36E-07	0.361	4.57E-12		IL13	0.109	3.60E-02	0.122	2.31E-02
	CD79A	0.156	2.54E-03	0.289	4.46E-08	Tfh	BCL6	0.127	1.47E-02	0.135	1.23E-02
	CD79B	1.148	4.36E-03	0.247	3.45E-06		IL21	0.141	6.53E-03	0.158	5.39E-04
	CD22	0.155	2.77E-03	0.244	4.40E-06	Th17	STAT3	0.13	1.21E-02	0.183	6.39E-04
	CIITA	0.251	9.31E-05	0.39	5.54E-14		IL17A	0.031	5.47E-01	0.046	3.90E-01
M1 Macrophage	INOS (NOS2)	0.001	9.87E-01	0.013	8.16E-01		RORγt	−0.157	2.45E-01	−0.231	1.50E-05
	IRF5	0.404	5.79E-16	0.413	1.11E-15	Treg	FOXP3	0.147	4.57E-03	0.233	1.25E-05
	IL6	0.01	9.86E-01	0.018	2.80E-02		CD25	0.234	5.00E-06	0.377	4.43E-13
	CD80	0.327	1.01E-10	0.459	2.31E-19		CCR8	0.319	3.23E-10	0.422	2.66E-16
	CD64 (FCGR1A)	0.294	7.72E-09	0.461	1.35E-19		STAT5B	0.225	1.24E-05	0.211	8.10E-05
	PTGS2	0.056	2.84E-01	0.189	4.02E-04		TGFB1	0.243	2.21E-06	0.348	2.94E-11
M2 Macrophage	CD163	0.039	4.51E-01	0.16	2.93E-03						
	CD206(MRC1)	-0.091	8.04E-02	−0.009	8.40E-01						
	VSIG4	0.073	1.61E-01	0.199	1.95E-04						
	MS4A4A	0.057	2.72E-01	0.2	1.79E-04						

Purity, correlation adjusted by purity; None, correlation without adjustment; TAM: tumor-associated macrophage; Th: T helper cell; Tfh: Follicular helper T cell; Treg: regulatory T cell; Cor: *R* value of Spearman’s correlation.

### Anti-Silencing Function 1B Predicts Immunotherapy Efficacy

**FIGURE 10:**
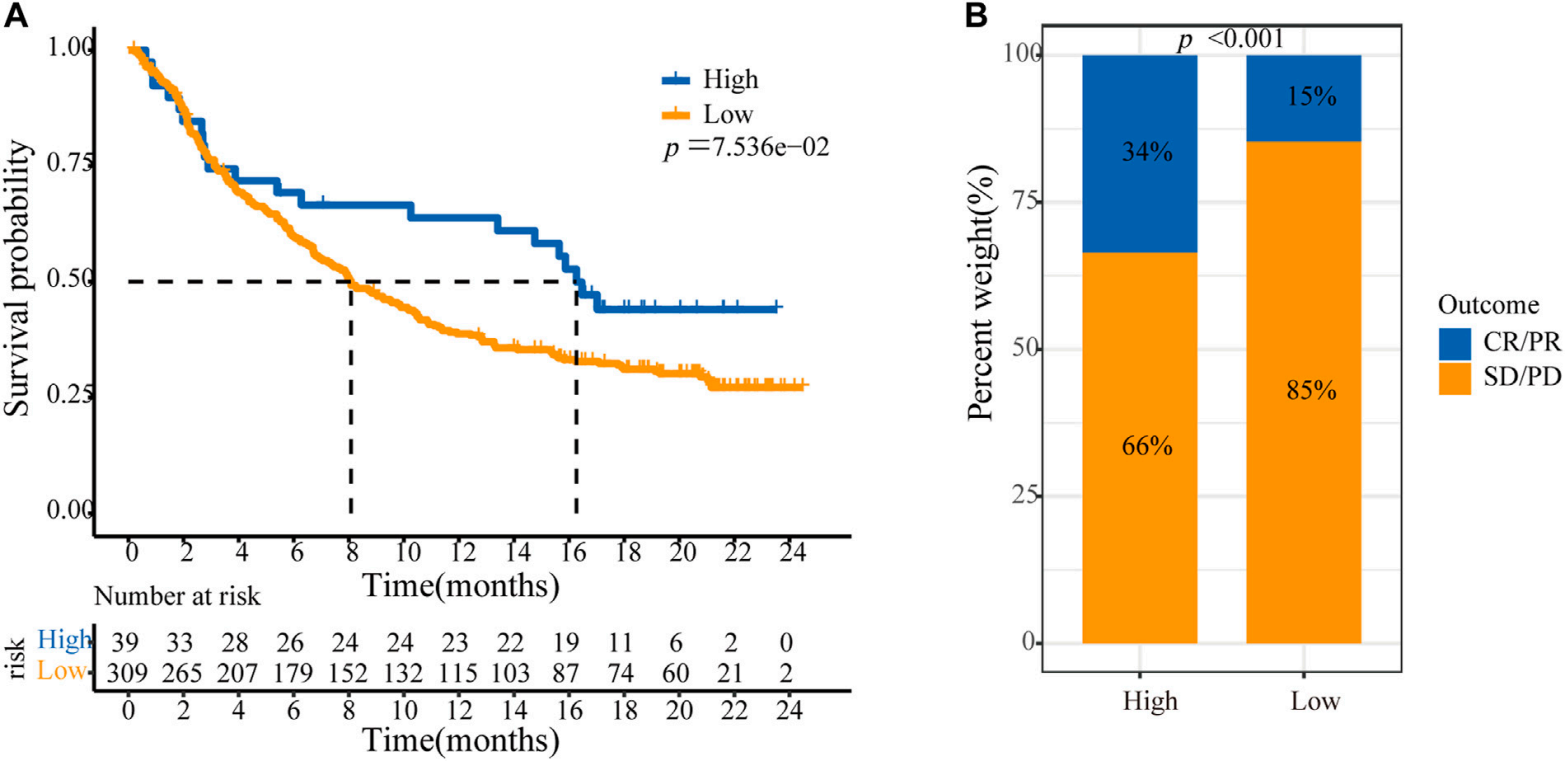
ASF1B predicts immunotherapy efficacy. **(A)** Kaplan–Meier analysis of OS according to the level of ASF1B expression among patients using immunotherapy; **(B)** The immunotherapy efficacy in ASF1B high and low expression groups. CR, complete response; PR, partial response; SD, stable disease; PD, progressive disease.

Because ASF1B was associated with immune cell infiltration in tumors and positively correlated with immune checkpoint coinhibitory molecules, we further explored the relationship between ASF1B and the efficacy of immunotherapy, and we also identified the value of ASF1B as a predictive marker. A transcriptome dataset (Imvigor210) of the treatment response data of patients who underwent anti-PD-L1 immunotherapy was retrieved to assess the ability of ASF1B to predict immunotherapy efficacy (Mariathasan et al., 2018). Kaplan-Meier analysis showed that although there was no significant difference, patients with high ASF1B had a relatively better outcome than patients with low ASF1B (*p* = 0.076) (Figure 10A). A higher objective response was observed in patients with high ASF1B expression than in patients with low ASF1B expression (34 vs. 15%, *p* < 0.001) (Figure 10B). These results suggested that ASF1B expression identifies patients who will respond to immunotherapy.

## Discussion

In the present study, by incorporating data from a public databases and our tumor center, we found that ASF1B expression was higher in tumor tissue compared to paracancerous normal tissues and that the high expression of ASF1B predicted poor outcome in HCC patients. Further systematic and comprehensive analysis of public databases revealed that ASF1B was also correlated with immune cell infiltration and activation in the tumor microenvironment of HCC and that it could predict the efficacy of immunotherapy.

The histone H3-H4 chaperone, ASF1, is a key factor for S-phase progression in the cell cycle in various organisms (Schulz and Tyler, 2006; Groth et al., 2007). Previous studies have highlighted the distinct functions of ASF1 isoforms (Abascal et al., 2013). It has been reported that ASF1B, rather than ASF1A, is critical for proliferation and has highly significant prognostic value in breast cancer and cervical cancer (Corpet et al., 2011; Han et al., 2018; Jiangqiao et al., 2019; Liu et al., 2020). ASF1B protein is increased by approximately 5.5-fold in tumor versus normal cells, and it is significantly correlated with the p60, p150, and Ki67 proliferation markers in breast cancer (Corpet et al., 2011). Disrupting ASF1B significantly suppresses tumor growth by G2/S stage cell cycle arrest and stimulates the apoptosis pathway. Furthermore, ASF1B levels, but not ASF1A levels, have a highly significant positive correlation with tumor size, tumor grade, and mitotic cell number. Thus, the expression of ASF1B has high prognostic value in breast cancer (Corpet et al., 2011). However, there exist no systematic and elaborated study on the expression of ASF1B in human HCC tissue and its association with the clinical prognosis of patients. Thus, in the present study, we evaluated ASF1B mRNA levels using different databases, and we evaluated protein levels by immunohistochemical analysis in HCC tumor and paracarcinoma tissues from patients treated at our clinical center. The results showed that the mRNA and protein levels of ASF1B were aberrantly high in HCC, and Western blotting of ASF1B protein levels in a normal liver cell line and tumor cell lines confirmed these results. In the tumor tissue, the expression of ASF1B was associated with tumor grade, T stage, and TNM stage. The survival analysis showed that high expression of ASF1B was closely associated with poor OS and PFS, indicating that ASF1B was an independent prognostic factor for HCC patients. These results were validated by independent samples collected from TCGA database and our center. Biological function analysis revealed that ASF1B was mainly correlated with the cell cycle but also with pathways that affected gene instability and cell proliferation, including DNA replication and gene repair in HCC. These results were consistent with studies addressing other cancers (Corpet et al., 2011; Han et al., 2018; Jiangqiao et al., 2019; Liu et al., 2020). The phosphatidylinositol 3 kinase (PI3K)/protein kinase B (Akt) signaling pathway plays a vital role in cell growth and proliferation, and has been observed to be dysregulated in various cancer types, including HCC (Zhu et al., 2021). It is reported that downregulation of ASF1B significantly decreased the phosphorylation levels of PI3K and protein Akt. Thus the PI3K/Akt pathway may be a possible mechanism underlying the carcinogenic effect of ASF1B (Han et al., 2018). In particular, it was observed that HCC patients in the high ASF1B group had a significantly higher frequency of TP53 and RB1 mutation, which are typical tumor suppressors, and their mutation leads to tumorigenesis and progression in HCC (Buendia, 2000; Khemlina et al., 2017). In addition, although the comparison of TMB was not different between the two subsets, MATH analysis revealed that the high-ASF1B group exhibited higher abundances of tumor heterogeneity, which is generally an indicator for poor clinical outcomes in multiple malignances, including HCC (Mroz et al., 2015; Ma et al., 2019; McDonald et al., 2019). However, the exact molecular events leading to cancer proliferation and poor prognosis in HCC have not yet been well elucidated, and further research is required. Altogether, ASF1B functions as an oncogene and is a potential diagnostic and prognostic biomarker in HCC.

Cell cycle dysregulation and immune escape are hallmarks of malignant tumors (Hanahan and Weinberg, 2011). Although neither phenomena is a single driver of tumor evolution, each stands for an important axis of potential therapeutic intervention. Surprisingly, several studies in recent years have revealed that these axes have crosstalk, and targeting cell cycle regulatory molecules improves the efficacy of tumor immunotherapy, thereby improving the response rate of immunotherapy (Goel et al., 2017; Deng et al., 2018; Greten and Korangy, 2018; Zhang H. et al., 2018; Zhou et al., 2018; Jin et al., 2019). Inhibition of CDK4/6 enhances tumor antigen presentation and effector T cell infiltration and activation, and it markedly suppresses the proliferation of regulatory T cells (Goel et al., 2017; Deng et al., 2018). Inhibition of cyclin-dependent kinase 9 (CDK9) leads to an increase in immune cell infiltration in the TIME, including CD45^+^ immune cells, CD3^+^ T cells, and activated dendritic cells (Zhang J. et al., 2018). It has been reported that ASF1B binds to CDK9 and inhibits its proteasome-mediated ubiquitination and degradation leading to the CDK9 protein stabilization (Liu et al., 2020). Thus, as a cell cycle regulator molecule, we comprehensively explored the relationship between ASF1B and the degree of immune cell infiltration in HCC using multiple databases including TCGA, TIMER, XCELL, QUANTISEQ, EPIC, CIBERSORT-ABS, CIBERSORT and ssGSEA. In the present study, we found that the ASF1B expression level was significantly correlated with the expression of cell-cycle modulators, which was accordance to previously published studies (Corpet et al., 2011; Liu et al., 2020). ASF1B expression was also significantly associated with immune checkpoints expression such as CD274, CTLA4, PDCD1 and so on. Meantime, we also found that cell cycle regulatory molecules were positively related to immune checkpoint molecules, further suggesting the close link between cell cycle and tumor immunity. Additionally, 13 cell-cycle modulators and nine immune checkpoints were significantly increased in the ASF1B-high group in HCC. We also found that ASF1B mRNA levels were positively correlated with the immune cells infiltration degree, such as CD4^+^ T cells, CD8^+^ T cells, B cells, neutrophils, macrophages, and DCs in HCC which was also confirmed by Zhan’s study. Furthermore, we explored the correlations of ASF1B expression with genetic markers of various immune cells after tumor purity adjusting. The results were in line with the above findings and showed that ASF1B expression was correlated with most immune cell markers in HCC. Zhan et al. (2021) has reported that downregulation of ASF1B inhibited the expression of CD86, CD8, STAT1, STAT4, CD68, and PD1 in HCC cells which further supported our results. However, we found that ASF1B was positively related to Treg cell infiltration and significantly associated with increased Treg markers, including CCR8, STAT5B, TGF-B1, and CD25. In addition, ASF1B was significantly positively correlated with T cell exhaustion markers (PD-1, CTLA4, LAG3, TIM-3, and TIGIT) in HCC. Moreover, the high ASF1B expression group correlated with impaired antitumor immunity, including the low activity of type II IFN response and type I IFN response, which support cytotoxic T lymphocytes by stimulating the maturation of dendritic cells and enhancing their capacity to process and present antigens (Miar et al., 2020). These results provided evidences that even though ASF1B positively correlates with tumor-infiltrating immune cells, such as CD4^+^ T cells and CD8^+^ T cells, infiltrating T cells in the tumor microenvironment are inactivated and exhausted. In addition, although TMB was not different between the ASF1B-high group and -low group, MATH analysis revealed that the high ASF1B expression exhibited higher abundances of tumor heterogeneity, which is generally associated with impaired immunity in multiple malignances (McDonald et al., 2019). Considered together, these data suggest that high ASF1B values might be correlated with immunosuppression in HCC. Previous studies have indicated that infiltrating immune cells and immune checkpoint expression in tumor sites influence prognosis and the response rate of immunotherapy (Calderaro et al., 2016; Kim et al., 2018). We found that patients with high ASF1B expression had higher patient response rates and achieved survival benefits from immune checkpoint inhibitors. The molecular mechanisms connecting cell cycle and immune surveillance, two of the most central processes in tumor biology, is still unclear. It is reported that inhibition of cell cycle regulators enhances tumour antigen presentation through stimulating production of type III interferons and finally increasing MHC class I molecules (Goel et al., 2017). And the CDK4/6 inhibitors promote PD-L1 expression by prevent the proteasome-mediated degradation inducing by Cullin3 SPOP E3 ligase (Zhang J. et al., 2018). In addition, the cell cycle block imposed on tumour cells is associated with cellular senescence, which promotes the secretion of cytokines and chemokines such as CCL5, CXCL9 and CXCL10, increase the infiltration and activation of T cells (Vilgelm et al., 2016). Exosomes, as the bridge between cells in tumor microenvironment, modulate the immune response (Zhang et al., 2021). Tumor microenvironment associated exosomes have the potential to serve as a breakthrough to clarify the connection between the cell cycle and immune surveillance. In addition to immune cells, other non-immune components in the tumor immune microenvironment can also affect tumor immunity, for example fibroblasts, myofibroblasts, endothelial cells, and extracellular matrix (Zhang J. et al., 2020; Guo et al., 2021). How ASF1B affects non immune cells remains to be explored. Therefore, as an important cell cycle regulator, how ASF1B modulates the TIME still needs to be verified by further extensive basic experiments.

The present study systematically reported the clinical significance of ASF1B and its effect on the TIME in HCC, but it also had several limitations. First, although we included data from our center to validate the expression and prognostic value of ASF1B, our results present limited evidence about the mechanism by which ASF1B upregulation affects the outcome of patients and the TIME with HCC. But we are currently conducting basic experiments *in vivo* and vitro to investigate how the aberrant elevation of ASF1B influences tumor behavior and the TIME in HCC. Second, the clinical data on immunotherapy administered to patients with HCC were unclear, which prevented us from performing a more detailed analysis. Thus, more clinical information needs to be collected on HCC patients applying immunotherapy to confirm these results.Third, integrating genomic, transcriptomic, proteomic, metabolomic and epigenomic datasets via multi-omics analysis could derive a deeper understanding of the development and progression of cancer (Sathyanarayanan et al., 2020; Su et al., 2020). More multi-omics data are needed to provide a comprehensive landscape of ASF1B roles in HCC.

## Conclusion

In summary, this study revealed that the expression of ASF1B was elevated in HCC tissues, and it could be a potential biomarker to predict HCC patient prognosis. In addition, high ASF1B expression is related to immunosuppressive tumor microenvironment characteristics with high expression of T cell exhaustive markers in HCC. However, high ASF1B expression predicts survival benefits from immune checkpoint inhibitors. Thus, these findings may be helpful in the management of patients in clinical practice as well as develop novel treatment strategies in the future to improve the sensitivity of immunotherapy.

## Data Availability

The original contributions presented in the study are included in the article/Supplementary Material, further inquiries can be directed to the corresponding author.
