# Supplementary figures and images for: ASF1B is a Promising Prognostic Biomarker and Correlates With Immunotherapy Efficacy in Hepatocellular Carcinoma
Source: Front Genet. 2022 Mar 10;13:842351. doi: 10.3389/fgene.2022.842351 (PMC8960381; doi:10.3389/fgene.2022.842351)

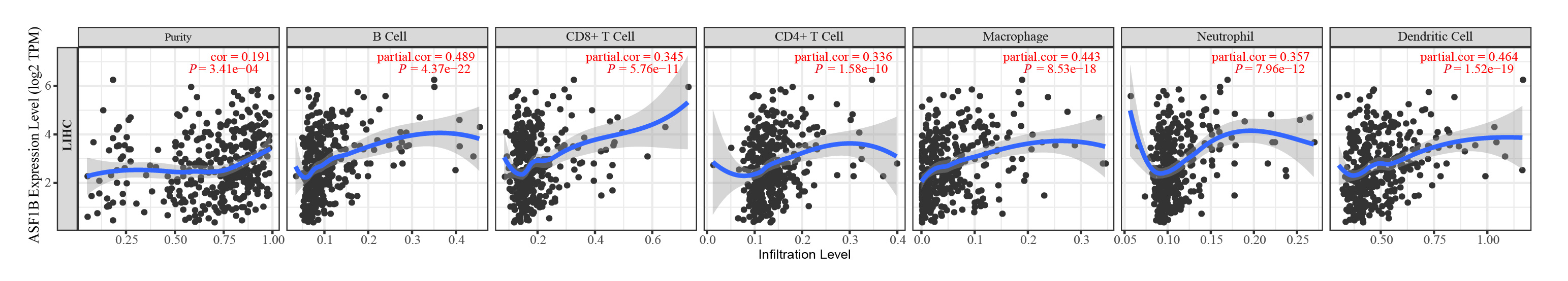

Supplement: Supplementary file 1 [file Image3.JPEG]

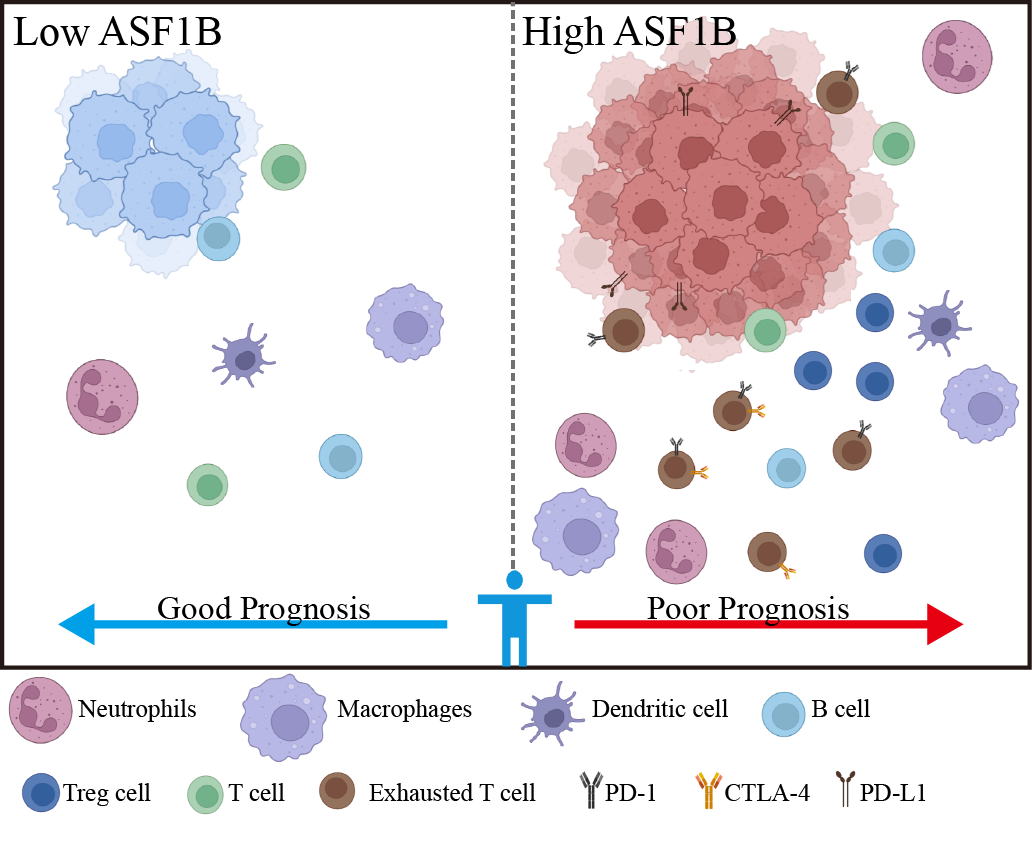

Supplement: Supplementary file 2 [file Image4.TIF]

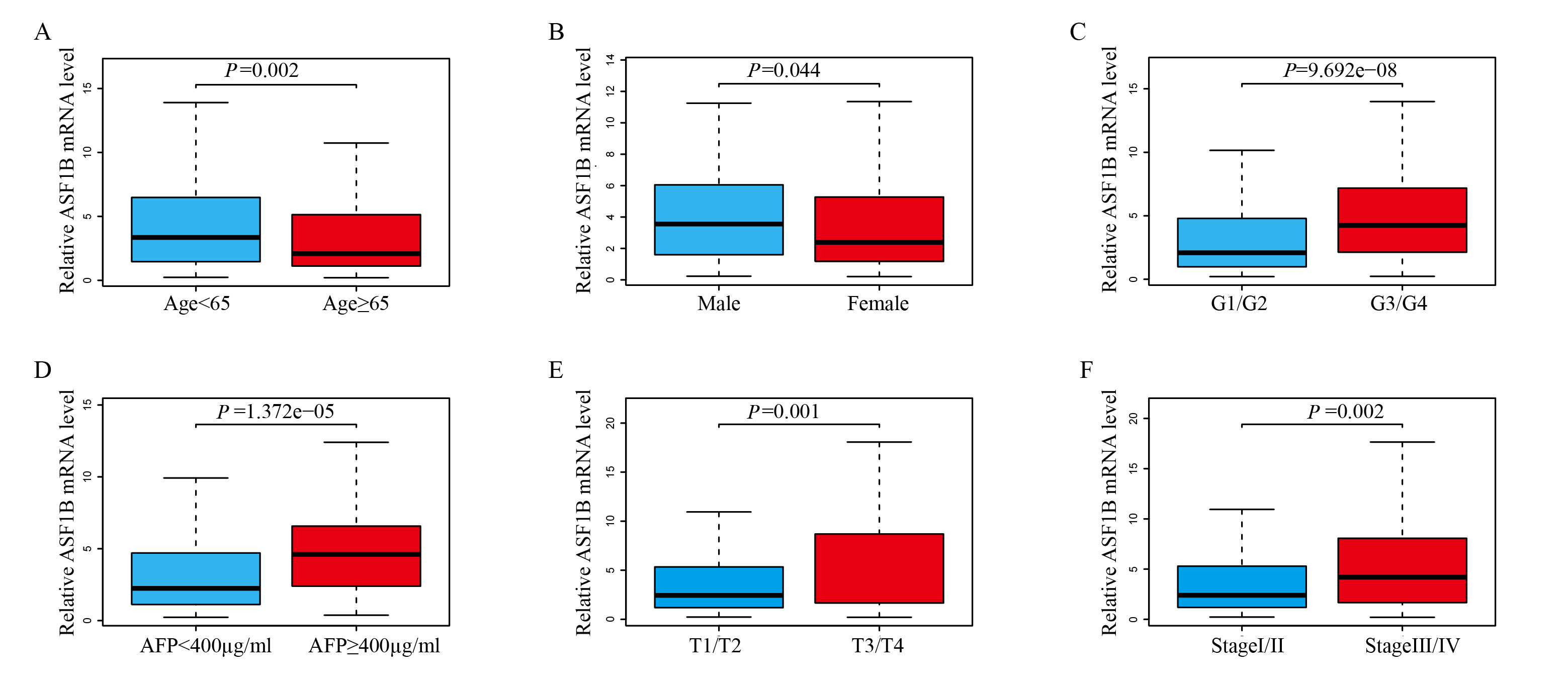

Supplement: Supplementary file 3 [file Image1.JPEG]

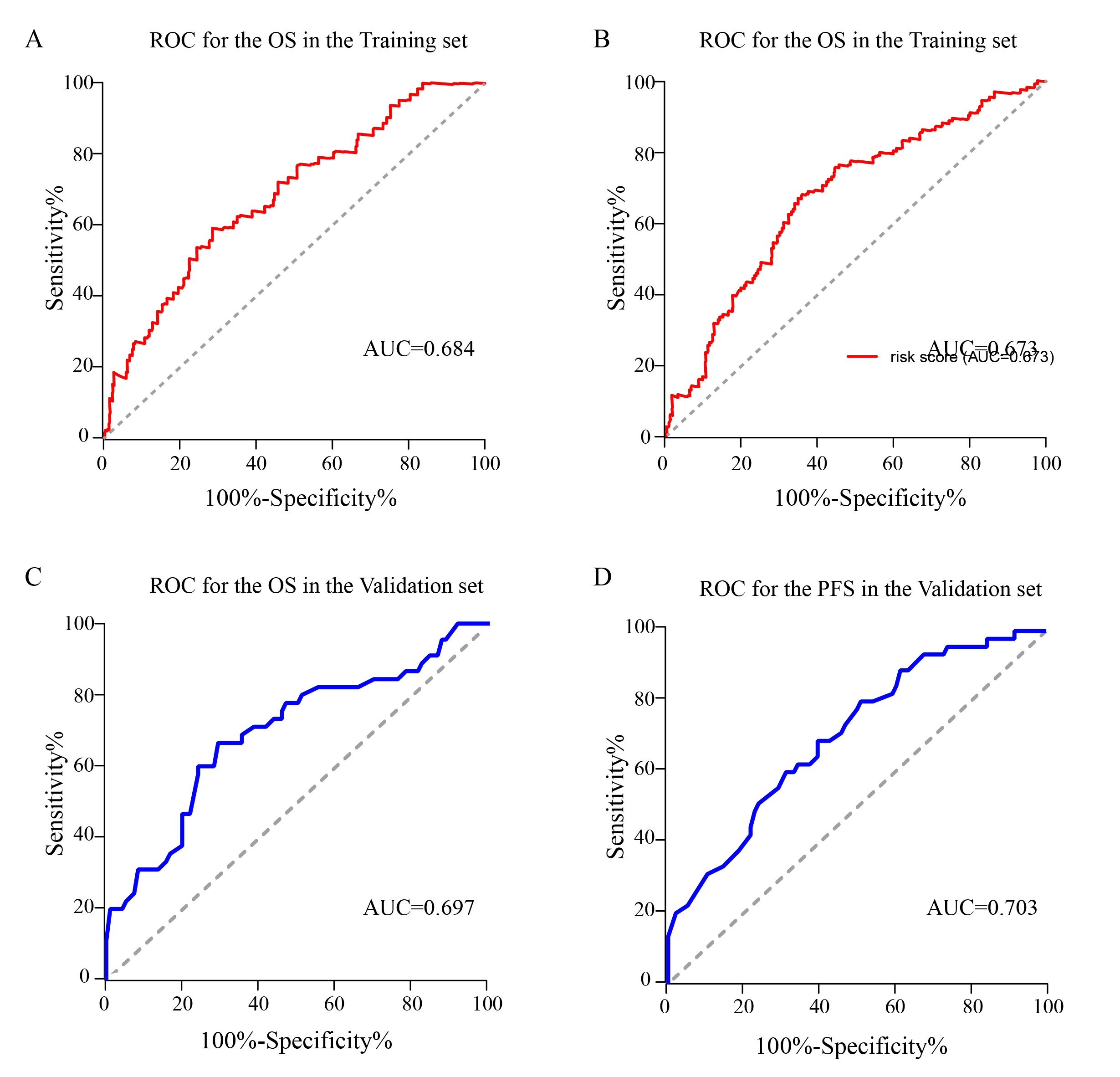

Supplement: Supplementary file 4 [file Image2.JPEG]
