# Supplementary material for: ASF1B is a Promising Prognostic Biomarker and Correlates With Immunotherapy Efficacy in Hepatocellular Carcinoma
Source: Front Genet. 2022 Mar 10;13:842351. doi: 10.3389/fgene.2022.842351 (PMC8960381; doi:10.3389/fgene.2022.842351)
Supplement: Supplementary file 5 [file Table1.DOC]

**Supplementary table 1.** The interrelationships between ASF1B expression and tumor immunological characteristics in HCC in multiple databases, including CIBERSORT, XCELL, CIBERSORT-ABS, QUANTISEQ, and EPIC.

| **Immune cell** | **cor** | ***P* value** |
| --- | --- | --- |
| B cell naive_CIBERSORT | -0.16188906 | 0.001757957 |
| B cell plasma_CIBERSORT | 0.143528223 | 0.005612978 |
| T cell CD4+ memory resting_CIBERSORT | -0.149567629 | 0.003884241 |
| T cell CD4+ memory activated_CIBERSORT | 0.205410229 | 6.73E-05 |
| T cell follicular helper_CIBERSORT | 0.266780172 | 1.83E-07 |
| T cell regulatory (Tregs)_CIBERSORT | 0.263551268 | 2.60E-07 |
| NK cell resting_CIBERSORT | -0.113536485 | 0.028774492 |
| Monocyte_CIBERSORT | -0.160650959 | 0.00190859 |
| Macrophage M0_CIBERSORT | 0.200975478 | 9.71E-05 |
| Macrophage M2_CIBERSORT | -0.172740326 | 0.000847785 |
| Mast cell activated_CIBERSORT | -0.102865081 | 0.04771586 |
| B cell plasma_CIBERSORT-ABS | 0.187412325 | 0.000283588 |
| T cell CD8+_CIBERSORT-ABS | 0.15412508 | 0.002916114 |
| T cell CD4+ memory activated_CIBERSORT-ABS | 0.205896543 | 6.46E-05 |
| T cell follicular helper_CIBERSORT-ABS | 0.35971723 | 8.94E-13 |
| T cell regulatory (Tregs)_CIBERSORT-ABS | 0.354188731 | 2.09E-12 |
| NK cell resting_CIBERSORT-ABS | -0.110136485 | 0.033949142 |
| NK cell activated_CIBERSORT-ABS | 0.24281016 | 2.22E-06 |
| Macrophage M0_CIBERSORT-ABS | 0.273660701 | 8.51E-08 |
| Macrophage M1_CIBERSORT-ABS | 0.173675364 | 0.000780834 |
| Macrophage M2_CIBERSORT-ABS | 0.13805568 | 0.007787519 |
| Myeloid dendritic cell resting_CIBERSORT-ABS | 0.111724488 | 0.031441195 |
| B cell_QUANTISEQ | 0.310930549 | 9.29E-10 |
| Macrophage M1_QUANTISEQ | 0.114988853 | 0.026779371 |
| Monocyte_QUANTISEQ | 0.252044612 | 8.75E-07 |
| NK cell_QUANTISEQ | -0.278488827 | 5.60E-08 |
| T cell CD4+ (non-regulatory)_QUANTISEQ | 0.173794901 | 0.000774225 |
| T cell CD8+_QUANTISEQ | 0.330879216 | 6.29E-11 |
| T cell regulatory (Tregs)_QUANTISEQ | 0.304833632 | 2.04E-09 |
| uncharacterized cell_QUANTISEQ | -0.223080484 | 1.52E-05 |
| T cell_MCPCOUNTER | 0.326010429 | 1.59E-10 |
| T cell CD8+_MCPCOUNTER | 0.184246504 | 0.000360471 |
| cytotoxicity score_MCPCOUNTER | 0.109779878 | 0.034579218 |
| NK cell_MCPCOUNTER | 0.122952646 | 0.01787386 |
| B cell_MCPCOUNTER | 0.243075925 | 2.33E-06 |
| Monocyte_MCPCOUNTER | 0.250071322 | 1.16E-06 |
| Macrophage/Monocyte_MCPCOUNTER | 0.250071322 | 1.16E-06 |
| Myeloid dendritic cell_MCPCOUNTER | 0.133784841 | 0.009930392 |
| B cell_XCELL | 0.277403707 | 5.57E-08 |
| T cell CD4+ memory_XCELL | 0.220078099 | 1.89E-05 |
| T cell CD4+ central memory_XCELL | -0.12254938 | 0.018205608 |
| T cell CD4+ effector memory_XCELL | -0.208957448 | 4.99E-05 |
| T cell CD8+ effector memory_XCELL | 0.14769395 | 0.004360433 |
| Class-switched memory B cell_XCELL | 0.211041813 | 4.18E-05 |
| Common lymphoid progenitor_XCELL | 0.463064426 | 4.07E-21 |
| Common myeloid progenitor_XCELL | -0.117161006 | 0.024017893 |
| Endothelial cell_XCELL | -0.513153148 | 2.61E-26 |
| Cancer associated fibroblast_XCELL | -0.183635647 | 0.000377379 |
| Granulocyte-monocyte progenitor_XCELL | -0.311015673 | 9.19E-10 |
| Hematopoietic stem cell_XCELL | -0.460865407 | 6.59E-21 |
| Macrophage_XCELL | -0.242071781 | 2.39E-06 |
| Macrophage M2_XCELL | -0.425014563 | 1.05E-17 |
| Mast cell_XCELL | 0.146464541 | 0.004700891 |
| B cell memory_XCELL | 0.116363764 | 0.025001452 |
| T cell NK_XCELL | 0.352342311 | 2.77E-12 |
| B cell plasma_XCELL | 0.106908619 | 0.039574913 |
| T cell gamma delta_XCELL | 0.182363847 | 0.000414977 |
| T cell CD4+ Th1_XCELL | 0.331825257 | 5.51E-11 |
| T cell CD4+ Th2_XCELL | 0.642272772 | 1.54E-44 |
| T cell regulatory (Tregs)_XCELL | -0.189758982 | 0.000236792 |
| stroma score_XCELL | -0.520216573 | 0 |
| microenvironment score_XCELL | -0.285127733 | 2.62E-08 |
| Cancer associated fibroblast_EPIC | 0.173029372 | 0.000830718 |
| Macrophage_EPIC | -0.354335816 | 2.81E-12 |
| uncharacterized cell_EPIC | 0.372999528 | 1.44E-13 |

**Supplementary Figure Legends**

**Supplementary Figure 1.** ASF1B expression is related to immune cell infiltration analyzed by TIMER database.

**Supplementary Figure 2.** ROC curve of ASF1B in the training set and validation set. AUC = area under the curve.

**Supplementary Figure 3.** The relationships between ASF1B expression and the patients’ clinical features in TCGA LIHC database.

**Supplementary Figure 4.** The hypothesis figure for the correlation between ASF1B expression and immune cell distribution.
